# Plasmids from Food Lactic Acid Bacteria: Diversity, Similarity, and New Developments

**DOI:** 10.3390/ijms160613172

**Published:** 2015-06-10

**Authors:** Yanhua Cui, Tong Hu, Xiaojun Qu, Lanwei Zhang, Zhongqing Ding, Aijun Dong

**Affiliations:** 1 School of Food Science and Engineering, Harbin Institute of Technology, Harbin 150090, China; E-Mails: tonghu1992715@gmail.com (T.H.); zhanglw@hit.edu.cn (L.Z.); dingzhongqing@hit.edu.cn (Z.D.); daj@hit.edu.cn (A.D.); 2 Institute of Microbiology, Heilongjiang Academy of Sciences, Harbin 150010, China; E-Mail: quxiaojuncyh@gmail.com

**Keywords:** lactic acid bacteria, plasmid, diversity, fermentation, probiotics

## Abstract

Plasmids are widely distributed in different sources of lactic acid bacteria (LAB) as self-replicating extrachromosomal genetic materials, and have received considerable attention due to their close relationship with many important functions as well as some industrially relevant characteristics of the LAB species. They are interesting with regard to the development of food-grade cloning vectors. This review summarizes new developments in the area of lactic acid bacteria plasmids and aims to provide up to date information that can be used in related future research.

## 1. Introduction

Lactic acid bacteria (LAB) are not a collective noun in classification, but are a heterogeneous group of Gram-positive, microaerophilic, non-sporulating and low G + C microorganisms which can ferment a range of carbohydrates to produce lactic acid [1,2]. LAB are commonly found in a variety of natural habitats, and are important industrial microbes that are used to produce a variety of industrial fermented food (dairy products, meat, wine, and silage *etc.*), macromolecules, enzymes, and metabolites. Some of them attract more attention from researchers as probiotics to maintain and regulate the human intestinal microflora [1,2].

Plasmid is a self-replication DNA molecule, which is non-attached to the cell chromosome and nuclear area DNA. The plasmids are not necessary genetic material for the survival of bacteria, but they often carry some special genes. They allow host strains to survive in a harsh environment and give the host strains greater competitiveness than other microorganisms, which are in the same environments [3]. A large number of plasmids were isolated and characterized from different sources of LAB as self-replicating extrachromosomal genetic materials [4]. Although most plasmids remain cryptic, some plasmids have been found that are associated with many important functions of LAB species, including (1) hydrolysis of proteins; (2) amino acid, citrate, and carbohydrate metabolism (e.g., lactose/galactose utilization, and oligopeptide transport); (3) production of bacteriocin, exopolysaccharide, and pigments; (4) resistance to antibiotic, bacteriophage, heavy metal, and other stress responses; and (5) DNA restriction-modification systems [4,5,6,7,8]. A variety of industrially relevant characteristics are encoded on the LAB plasmids, including the degradation of casein, acidification by lactic acid, and production of flavor compounds, which contribute to the desired flavor and texture of the fermentation product and to optimal growth of strains in milk [6,7,8,9,10]. These plasmids confer adaptive advantages improving the growth and behavior of their host cells. The latter are more suitable to be used in the food industry as starter cultures. Meanwhile, LAB plasmids are widely used for construction of expression systems of LAB, which is an effective means of enhancing the industrial applicability of LAB and minimizing their negative effects [7]. Identification, classification, construction, and application of LAB plasmids have attracted considerable scientific and technological attention. At present, there are over 400 LAB plasmids which have been isolated and studied [3,4,5,6,7,8,9,10]. This review presents information concerning the diversity of LAB plasmids, their replication mechanisms, structures, functions, and applications. The article will focus on the food LAB plasmids.

## 2. Diversity and Similarity of Plasmids from LAB

Plasmids are most commonly present in different LAB species, involved in 11 genera, including *Bifidobacterium*, *Brevibacterium*, *Enterococcus*, *Lactobacillus*, *Lactococcus*, *Leuconostoc*, *Oenococcus*, *Pediococcus*, *Streptococcus*, *Tetragenococcus*, and *Weissella*. LAB plasmids are extremely diverse in terms of size (0.87 kb to more than 250 kb), copy number (from 1 to more than 100 plasmids per cell), and phenotypes conferred to their hosts [5,6,8,9,10,11,12].

### 2.1. Plasmids of Genus Lactobacillus

*Lactobacillus* is the largest genus of the LAB group, with over 100 species in total [1,13]. They are widely distributed in variety of natural habitats including the oral, vaginal, and intestinal regions of many animals, fermented food, wine and other alcoholic beverages [1,2]. To date, 22 species of *Lactobacillus* have been identified that contain plasmids. *Lactobacillus* plasmids vary widely in size (from 1.81–242.96 kb), number (from 1–10 different plasmids in a single strain), and gene content [6,14,15].

Up to date, *Lactobacillus plantarum* contains the largest plasmids in the genus *Lactobacillus*. It is commonly isolated from plant material, and the gastrointestinal tract of animals [16,17]. This organism is used in the production of fermented foods, namely sauerkraut, kimchi and sourdough bread *etc.* [16]. It is also of interest as a probiotic to maintain and regulate the human intestinal microflora [18,19]. *L. plantarum* often harbors one or more natural plasmids of various sizes. *L. plantarum* strain 16 harbors the largest plasmid complement reported for this species to date, 10 plasmids (pLp16A–pLp16L), which range in size from 6.46–74.08 kb [20]. The strain 16 possesses broad-spectrum antifungal activities and has the potential to be used as a biopreservative agent to improve the shelf life of foods.

At present at least 56 plasmids from *L. plantarum* have been sequenced (Table S1, http://www.ncbi.nlm.nih.gov). Although most *L. plantarum* plasmids are cryptic, several plasmids encode some important properties, including antibiotic resistance [21,22,23,24], exopolysarccharide biosynthesis [20], chloride or potassium transport [20,25], bacteriophage resistance [26], as well as bacteriocin production [27].

Many small plasmids from *L. plantarum* replicate via the rolling-circle replication (RCR) mechanism, while some large plasmids, for example pMD5057, pWCFS103, and pST-III, are predicted to replicate via the theta mechanism [15,22,25,28,29,30,31,32,33]. Studies indicated that a rolling-circle plasmid pMRI 5.2 from a potentially probiotic strain *L. plantarum* BFE 5092, had two different plasmid-encoded replication initiation proteins from different replicon families, *i.e.*, pMV158 and pC194 family [30]. This result suggests that the genes for these replication initiation proteins may have originated from different plasmids [30].

Megaplasmids of sizes ranging from 120–490 kb are found in *Lactobacillus salivarius*, *Lactobacillus acidophilus*, *Lactobacillus hamster*, *Lactobacillus intestinalis*, *Lactobacillus kalixensis*, *Lactobacillus ingluviei*, and *Lactobacillus equi*, including pMP118 (242.44 kb), pHN3 (242.96 kb), pWW1 (194.77 kb) [14,34,35,36,37,38] *etc.* Studies indicated that megaplasmid pMP118 from *L. salivarius* UCC118 was involved in rhamnose, sorbitol, and ribose utilization [35]. At the same time, it was speculated that pMP118 was likely to contribute to host colonization or probiotic properties [34].

A large number of vectors based on native plasmids from *Lactobacillus* strains have been developed in the past 20 years [6,7,39,40,41,42,43,44,45,46,47,48,49,50]. The most common expression system is the pSIP expression system, which is derived from *Lactobacillus sakei*, and is based on the regulatory system of antimicrobial peptides sakacin A or sakacin P and the quorum sensing mechanism [39,40]. The pSIP vectors have been used to express high amounts of heterologous proteins in lactobacilli, such as β-glucuronidase, amino peptidase, amylase, and β-galactosidases *etc.* [39,40,41,42]. However, due to the use of an erythromycin antibiotic resistance gene as selection marker, the potential of the pSIP system for food applications has been limited.

Therefore several new food-grade selection markers and corresponding expression systems have been developed [43,44]. Nguyen *et al.* developed a food-grade system for inducible gene expression in *L. plantarum* using an alanine racemase-encoding selection marker, which is a complementation selection marker, and can be used only with *alr* deletion mutants of *L. plantarum* [43]. A novel vector pM4aB for LAB was developed using a bile salt hydrolase gene from *L. plantarum* as a potential food-grade selection marker [44]. The vector pM4aB contains replicon of *L. plantarum* plasmid pM4, and it has expressed a catalase gene from *L. sakei* in *L. paracasei* [31,44]. Recently, a novel expression system for *L. plantarum* has been developed, which is based on the manganese starvation-inducible promoter from the specific manganese transporter of *L. plantarum* NC8 [45]. Its advantages are that no addition of an external inducing agent is required, and additionally, no further introduction of regulatory genes is necessary.

A series of *Escherichia coli*/*Lactobacillus* shuttle vectors was constructed, which was useful as a gene manipulation tool for LAB [46,47,48,49,50]. The shuttle vector pLES003 contains a replication origin from *Lactobacillus brevis* plasmid pLB925A03, a ColE1 origin, and the multi-cloning site from pUC19 [46]. pLES003 can replicate in cells of *E. coli*, *L. brevis*, *L. plantarum*, *Lactobacillus helveticus* and *Enterococcus hirae* [46]. The shuttle vector pGYC4α was constructed based on the RCR plasmid pYC2 from *L. sakei* BM5 isolated from kimchi [47]. pGYC4α expressed α-amylase from *Bacillus licheniformis* in *E. coli*, *Lactococcus lactis* MG1363, *L. lactis* MG1614, *Leuconostoc citreum* C16, and *Leuconostoc mesenteroides* C12 [48]. Replicons of theta-type-replicating plasmids pRCEID2.9 and pRCEID13.9 from Lactobacillus casei strain TISTR1341, have been used to develop *E. coli*/*L. casei* compatible shuttle vectors, which were stably maintained in different genetic backgrounds [49]. Recently, a novel plasmid pMC11 was isolated from *L. casei* MCJ, which is a starter culture for a traditional yoghurt product in China [50]. It contains two distinct replicons both of which replicate via a theta replication mechanism. The shuttle vectors pEL5.7 and pEL5.6 were constructed with two replicons of pMC11, respectively [50]. Meanwhile, the corresponding expression vectors pELX1 and pELX2 successfully expressed a green fluorescent protein in different *Lactobacillus* species [50]. These shuttle vectors provide efficient genetic tools for DNA cloning and heterologous gene expression in LAB.

### 2.2. Plasmids of Genus Lactococcus

The genus *Lactococcus* is widely used in food fermentation [51]. The member of *Lactococcus* genus *L. lactis* remains the best characterized *Lactococcus* species with regards to physiology and molecular genetics [52]. It is found in diverse environments, e.g., plant and animal habitats [53]. *L. lactis* is one of the most extensively used starter cultures in the LAB group, for production of various fermented dairy products, for example cheese, sour cream and fermented milks *etc.* [53,54].

*L. lactis* strains carry plenty of plasmids [9,10]. Most *L. lactis* strains usually contain 4–7 plasmids, which range in size from 0.87 kb to more than 80 kb [11,55]. The plasmid profile analysis of *Lactococcus* strains has shown that 150 dairy starter cultures (90 *Lactococcus lactis* subsp. *cremoris*, 30 *Lactococcus lactis* subsp. *lactis*, and 30 *Lactococcuslactis* subsp. *lactis* biovar *diacetylactis*) gave an average of seven plasmids per strain, ranging from 2–14. In the plant strains, the average was less two plasmids per strain, ranging from 0–4. The results showed that industrial dairy strains possessed a higher average plasmid complement than non-dairy strains. Meanwhile, the former strains contained a greater abundance of plasmids smaller than 10 kb [52].

Plasmids of *L. lactis* are involved in essential functions, e.g., bacteriocin production [56,57,58,59], cadmium resistance [60,61], antibiotic resistance [62], as well as in industrially relevant and significant characteristics, namely citrate utilization [63], casein utilization [55,64], lactose utilization [55,63,64,65], oligopeptide transport [63,65,66], orotate transport [67], cation transport [62,65,68], stress response and adaption [62,63,68,69], exopolysaccharide production [70], anti-phage restriction/modification systems [61,62,63,71,72,73,74], folate biosynthesis [65], proteolysis [65,75], and conjugal transfer [76].

Some plasmid-encoding characters can be used as selectable markers for the development of vectors, which are needed for cloning and to stably maintain the plasmid during bacterial growth, such as nisin resistance, cadmium resistance, zinc resistance, and thermo stability *etc.* [60,66,77,78]. Most of the lactococcal vectors rely on antibiotic resistance markers, for example erythromycin and chloramphenicol [7]. However, the use of antibiotics limits the application of vectors with antibiotic resistance markers, especially in the food industry and practical vaccines production [79]. Therefore some food-grade selectable marker systems are being developed, which are based on the complementation of auxotrophs and mutations in metabolic genes [80,81,82,83,84,85]. The use of bacteriocins as selecting agents and their cognate immunity genes as selectable marker genes have also been explored as food-grade cloning strategies [83,84,85]. Some bacteriocins have been used, e.g., nisin, lacticin 481 and lacticin 3147 [83,84,85]. Recently, bacteriocin lactococcin 972 (*Lcn972*) gene cluster has been used as a food-grade post-segregational killing system to stabilize recombinant plasmids in *L. lactis* in the absence of antibiotics [59]. Once the *Lcn972* recombinant plasmid is inside the cells, it is maintained without any obvious deleterious consequences for the cells and antibiotic pressure is no longer needed, providing a useful tool for the development of safer and more sustainable biotechnological applications of *L. lactis* [59].

Studies on lactococcal plasmids have attracted considerable interest. At present, approximately 86 lactococcal plasmids have been completely sequenced, and 14 plasmids have been partially sequenced (Table S2, http://www.ncbi.nlm.nih.gov).

**Figure 1:**
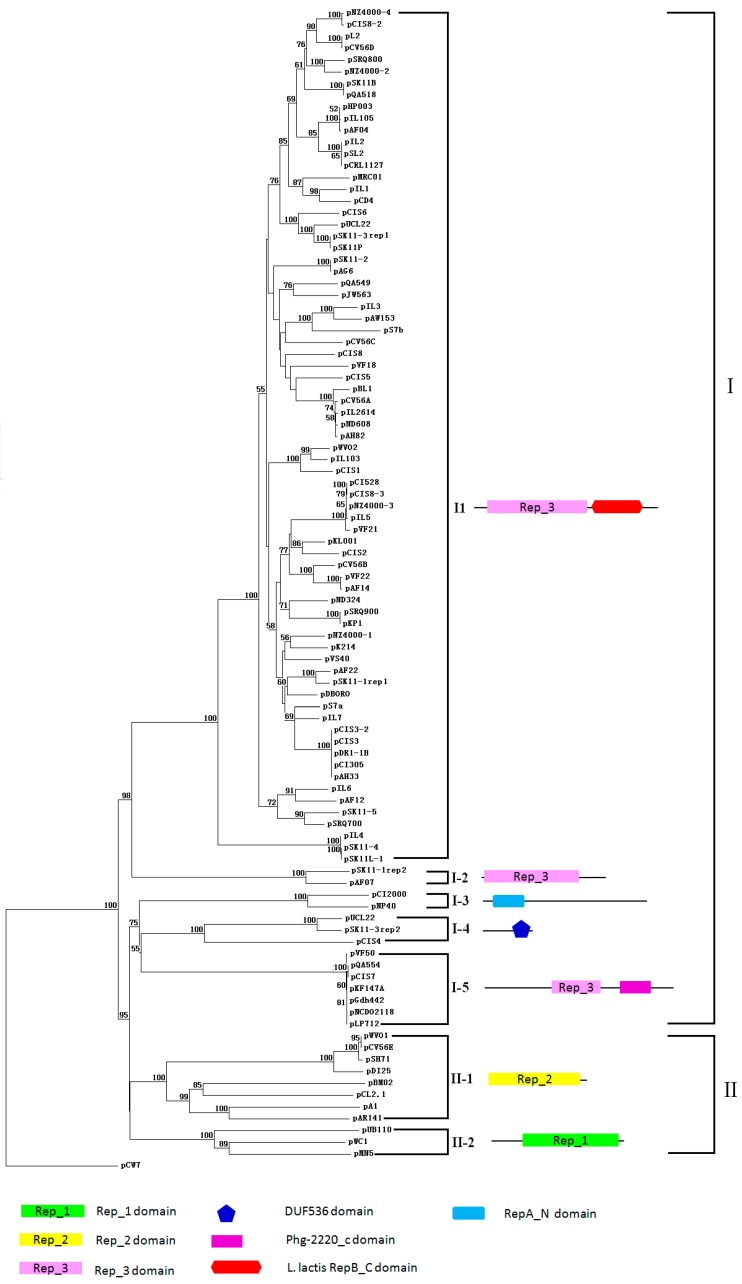
A Neighbor-joining tree was constructed with the ClustalW using Rep proteins from *Lactococcus lactis* plasmids. Bootstrap values for a total of 100 replicates are shown at the nodes of the tree. The plasmid pCW7 (a pT181 type RCR plasmid) was used as an out-group. Bar 1% sequence divergence.

For a comparative analysis of replication proteins encoded by lactococcal plasmids, a phylogenetic analysis of 96 Rep proteins from lactococcal plasmids and 3 Rep proteins from pUB110, pA1, pCW7 was performed, and it categorized lactococcal plasmids into two groups based on their Rep proteins (Figure 1).

Most Rep proteins (87/99) belong to group I. Subgroup I-1: includes 73 Rep proteins from lactococcal plasmids. This is the most commonly detected replicon in lactococcal plasmids. Members of this subgroup exhibit a conserved Rep_3 domain (Pfam database, PF01051) and *L. lactis* RepB_C domain (Pfam database, PF06430).The latter domain is found in the C-terminal region of RepB proteins from *L. lactis*. Some members of this subgroup, pCI305 [86], pWVO2 [87], pW563 [88], and pCD4 [89], are shown experimentally to replicate via the theta replication, suggesting that these plasmids in subgroup I-1 may replicate via theta replication mechanism.

Subgroup I-2 encompasses two Rep proteins from pSK11-1 and pAF07, which contain the Rep_3 domain (Pfam database, PF01051). Comparing with members of subgroup I-1, this subgroup has not *L. lactis* RepB_C domain.

Subgroup I-3 contains Rep proteins from pCI2000 and pNP40. This subgroup shows a conserved RepA_N domain (Pfam database, PF06970) at the N-terminal region, which is noted to contain a helix-turn-helix motif. This is a remarkable feature of pLS32-type theta replication proteins [90]. Meanwhile, a 40-bp sequence directly repeated two and three quarter times is present on Rep proteins of pCI2000 and pNP40 [73,78,91]. Such repeats, also termed iterons, are common elements of the origin of replication (*ori*) for many theta replicating plasmids [92].

Subgroup I-4 contains 3 Rep proteins with a special DUF536 domain (Pfam database, PF04394) at C-terminal, which is found in several bacterial proteins of unknown function that may be involved in a theta-type replication mechanism. One of three plasmids, pUCL22, was shown to replicate via theta replication mechanism, suggesting that the replication mode of other plasmids in subgroup I-4 (pSK11-3 and pCIS4) may follow theta replication [93,94].

Subgroup I-5 consists of Rep proteins of pVF50, pQA554, pCIS7, pKF147A, pGdh442, pNCDO2118, and pLP712, containing a conserved Rep_3 domain (Pfam database, PF01051) and a conserved Phg_2220_c domain (Pfam database, PF09524). The latter domain is found exclusively in bacteriophage and in the bacterial prophage region, but the functions of this domain are unknown. It was confirmed that the region upstream of *repA* of pGdh442 contained motifs characteristic of the replication origins of lactococcal theta-type replicons [66]. Therefore this result suggests that members of subgroup I-5 replicate via theta replication. Interestingly, the plasmids pVF50, pQA554, pCIS7, and pLP712, are isolated from dairy niche, however, pGdh442, pKF147A, and pNCDO2118 originated from plant niche [55,64,66,68,69,95,96], suggesting there is extensive horizontal gene transfers among these plasmids.

Group II contains 9 Rep proteins from lactococcal plasmids, Rep protein of pA1 (pE194/pMV158 family of RCR plasmid), and Rep protein of pUB110 (pC194-type of RCR plasmid). All plasmids of this group replicate by means of rolling circle, which involves the synthesis of ssDNA intermediates. Most RCR lactococcal plasmids belong to the pE194/pMV158 family, except to pWC1 and pMN5, which are pC194-type of replicons [58,62,97,98,99,100,101,102,103].

RCR lactococcal plasmids exhibit a broad host range, being able to replicate a range of Gram-positive and Gram-negative bacteria, e.g., *E. coli*, which is the most widely studied prokaryotic model organism and used widely for genetic operation in the fields of biotechnology and microbiology [99,101,104]. A series of wide host range vectors (*i.e.*, pCK-, pNZ-, pFIAV-series) were constructed from lactococcal RCR plasmids, and used for transformation LAB and other hosts [7,105].

However, RCR plasmids are incompatible with other RCR plasmids, so a single lactococcal strain never contains more than one RCR plasmid [99]. At the same time, RCR plasmids have been shown to be structurally and segregationally unstable due to their single-stranded mode of replication, which can produce accumulation of ssDNA intermediates [106]. RCR plasmids have a limited replicon size (10 kb), therefore cloning vectors based on the replicons of RCR plasmids can only be used to clone relatively small DNA fragments due to their structural and segregational instability with large inserts [106]. The above-mentioned factors limit the application of the derived vectors of RCR plasmids.

To solve vector stability problems, the focus has been on developing vectors based on theta-replicating plasmids [7]. In contrast to RCR plasmids, theta-type plasmids replicate by means of a double-stranded rather than a single-stranded replication intermediate, which results in better structural stability, allowing for the insertion of large heterologous DNA fragments. This property is useful for the construction of cloning vectors. Meanwhile, lactococcal theta derived vectors are compatible with endogenous RCR plasmids. Most theta-replicating lactococcal plasmids are members of a family of highly related, compatible replicons, so they can coexist in the same *L. lactis* strain [107]. It would be a great advantage to have a series of compatible vectors available.

The pathogen *Lactococcus garvieae* 21881 harbors five circular plasmids, namely pGL1 (4.54 kb), pGL2 (4.57 kb), pGL3 (12.95 kb), pGL4 (14.01 kb), and pGL5 (68.8 kb). The plasmid pGL2 replicates via the rolling circle mechanism, however, the other four plasmids are theta-replicating [108]. The plasmids pGL1, pGL2, and pGL5 encode putative proteins related with the synthesis, secretion, and immunity of bacteriocin. The plasmid pGL5 harbors genes (*txn*, *orf5* and *orf25*) encoding proteins that could be considered putative virulence factors [108].

### 2.3. Plasmids of Genus Pediococcus

The genus *Pediococcus* possesses a homofermentative metabolism, and is commonly found in a variety of natural habitats, including the surface of plants and fruits, and fermented food (e.g., cured meat, raw sausages, and marinated fish *etc.*). It is applied industrially in food fermentations, and used for biotechnological processing and preservation of foods [109].

Pediococci species, mainly *Pediococcus pentosaceus* and *Pediococcus acidilactici*, harbor many different plasmids, ranging in size from 1.82–190 kb. Some plasmids encode a variety of traits, such as utilization of raffinose and sucrose [110,111], antibiotic resistance [112,113], as well as bacteriocin production and immunity [114,115,116,117,118,119].

Currently, there are a number of pediococcal plasmids which have been completely sequenced. pEOC01, a plasmid (11.661 kb) from *P. acidilactici* NCIMB 6990, encodes multidrug resistance (*i.e.*, clindamycin, erythromycin, and streptomycin). The plasmid contains a streptomycin resistance gene *aadE* gene which holds 100% identity to an *aadE* gene found in Gram-negative bacterium *Campylobacter jejuni* plasmid. This observation is significant in that it provides evidence for recent horizontal transfer of streptomycin resistance from a lactic acid bacterium to a Gram-negative intestinal pathogen and as such infers a role for such plasmids for dissemination of antibiotic resistance genes possibly in the human gut [120].

*P. pentosaceus* can be isolated from a variety of plant materials and bacteria-ripened cheeses. This organism is used as an acid producing starter culture in the fermentation of some sausages, cucumbers, green beans, soy milk, and silage. It is also a typical component of the adventitious microflora of most cheese varieties during ripening. Some strains have been reported to contain several resident plasmids that render the bacterium capable of fermenting some sugars (raffinose, melibiose, and sucrose), as well as producing bacteriocins [114,117,118,121,122].

Pediocin PA-1/AcH, an anti-listerial class IIa bacteriocin, is produced primarily by several pediococcal strains, including *P. acidilactici* strains PAC1.0, H, E, F, M, K10, HA-6111-2, HA-5692-3, MM33; *Pediococcus parvulus* ATO34, ATO77 and *P. pentosaceus* FBB61 [119]. The genetic determinants of the biosynthesis of pediocin PA-1/AcH are located within a plasmid-borne operon cassette in all producing LAB strains examined to date, including pSRQ11 (9.4 kb), pSMB74 (8.9 kb), pATO77 (3.509 kb), pS34 (3.509 kb), pWHE92 (3.510 kb), and pMD136 (19.5 kb) *etc.* [116,117,118,119,123,124,125,126,127].

Pediocin PA-1/AcH is also synthesized by other LAB, except pediococcal strains, including *L. plantarum* WHE92 [128], *L. plantarum* DDEN 11007 [129], *L. plantarum* Acr2 and *E. faecium* Acr4 with plasmid-coding [130,131]. The plantaricin 423 from *L. plantarum* 423, is encoded in plasmid pPLA4. The operon structure of plantaricin 423 is similar to pediocin PA-1/AcH from *P. acidilactici*. The *plaC* and *plaD* genes are virtually identical to *pedC* and *pedD* of the pediocin PA-1 operon, as well as *coaC* and *coaD* of the coagulin operon [27]. The antilisterial bacteriocin coagulin, produced by *Bacillus coagulans* I_4_, has an operon which shows high similarity with the pediocin operon [132]. The results show horizontal gene transfer among these plasmids from different species. It has been shown that the plasmids responsible for production in *P. acidilactici* H can be transferred intragenerically by conjugation [133]. Recently the flanking regions of the pediocin PA-1/AcH (pediocin PA-1) operon were characterized in order to evaluate mobile genetic elements in intergeneric and interspecific pediocin producing LAB [130]. Studies showed that ISLpl1, tyrosine recombinase and mobilization regions were found, which were known to be associated with transfer of genes linked to bacteriocin production, antibiotic resistance, and sugar utilization [130].

**Figure 2:**
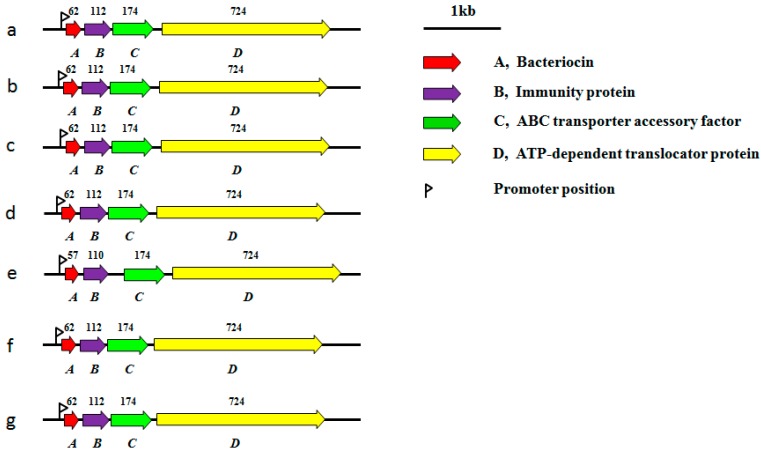
Organization of the gene clusters of pediocin and related bacteriocins from different plasmids. (**a**) pS34 from *P. pentosaceus* (*Pediococcus pentosaceus*) S34 (NG_035883.1); (**b**) pATO77 from *P. parvulus* (*Pediococcus parvulus*) ATO77 (NG_035882.1); (**c**) pSMB74 from *P. acidilactici* (*Pediococcus acidilactici*) H (NC_004832.1); (**d**) pWHE92 from *L. plantarum* WHE92 (NG_035884.1); (**e**) pPLA4 from *L. plantarum* 423 (AF304384.2); (**f**) pI4 from *B. coagulans* I_4_ (NG_035346.1); and (**g**) pEnt4 from *E. faecium* Acr4 (NG_041274.1). Open reading frames (ORFs) encoding the related proteins are marked with a different color. The number of amino acid residues within each encoded protein is shown above the corresponding ORF.

In several strains, the sizes and organization of the various pediocin-encoding plasmids are similar, including pATO77 from *P. parvulus* ATO77, pS34 from *P. pentosaceus* S34, and pWHE92 from *L. plantarum* WHE92 [119,123,125,126,127,128,134,135] (Figure 2). Structure, immunity and secretion system genes are linked together in the operons, and the promoter sequences are the same. Pediocin can be used as a selection marker in cloning vectors, therefore, plasmids that carry the genes for its production, can be used for development of food-grade cloning vectors [122].

*Pediococcus claussenii* is a common beer spoilage organism. *P. claussenii* ATCC BAA-344T contained eight plasmids (pPECL-1 to pPECL-8) that encode a variety of traits, including drug resistance, conjugation protein, the toxin-antitoxin (TA) system, and bacteriocin *etc.* [136]. From the point of view of beer spoilage, several genes are interesting, including the already-known beer spoilage-associated gene *horA* found on pPECL-8. The plasmids pPECL-6 and pPECL-7 are not circularized due to repetitive transposon regions found in both plasmids, making PCR-based gap closing very difficult. The overall G + C content of the genome is 36.8%, whereas that of the plasmids ranges from 34.9%–42.5%. The results show that there is a horizontal gene transfer in this bacterium.

### 2.4. Plasmids of Genus Enterococcus

Enterococci are Gram-positive cocci that can survive harsh conditions in nature. They can be found in soil, water, and plants. Some strains are used in the manufacture of foods, and improved the typical taste and flavor of many foods (such as cheeses and sausages) by their proteolytic and lipolytic activities [137,138]. However, some enterococci are opportunistic pathogens causing serious human and animal infections because of their virulence genes and resistance to antibiotics [138,139].

Enterococci harbor abundant plasmids, some of which are characterized encoding antibiotic resistance (e.g., erythromycin, tetracycline, gentamicin, teicoplanin, and macrolide-lincosamide-streptogramin B antibiotics) [12,140,141,142,143,144], bacteriocins (e.g., enterocin 1071A, enterocin 1071B, enterocin I, enterocin J, enterocin Q, bacteriocin 51, and lactococcin) [145,146,147,148,149] as well as virulence factors, namely aggregation substance protein [150,151], surface exclusion protein [152], extracellular surface protein [144,152], cell wall surface anchor family protein [149], cytolysin [144], toxin [153], and sex pheromone [154]. The abilities of enterococci to produce bacteriocins and to adapt to different environmental conditions are important characteristics for the food industry [138,155].

The plasmid pAR6 from *E. faecium*, contains a newly characterized heat shock promoter (P_hsp_), which was found to regulate the expression of α-crystallin heat shock protein (hsp) in the plasmid [156]. P_hsp_ was used for construction of a novel *Lactobacillus* vector pAR1801, which can replicate in *L. plantarum* and *L. lactis* [156].

### 2.5. Plasmids of Streptococcus thermophilus

*Streptococcus thermophilus* is the only streptococcal species used in food fermentations, and has been widely used as a yogurt manufacturing starter culture to produce yogurt together with *Lactobacillus delbrueckii* subsp. *bulgaricus* for thousands of years throughout the world.

Like *L. delbrueckii* subsp. *bulgaricus*, *S. thermophiles* strains carry very few plasmids [7,157]. Most plasmids of *S. thermophiles* are cryptic, as no apparent phenotypic traits seem to be associated with their presence. Some plasmids have been found to encode small heat shock proteins, including pER341 [158], pCI65st [159], pND103 [160], pST04 and pER1-1 [161], pt38 [162], pER7, pER16, pER26, pER35, pER36, and pER41 [163,164], pK1002C2, and pK2007C6 [165].

Some researches indicated that these small heat shock proteins were induced by elevated temperatures and low pH, and expression of these proteins increases thermo- and acid resistance of the strains that carry heat shock proteins [161,164]. Therefore the promoter of heat shock protein gene *hsp16.4* of pER341 is under investigation for potential use in temperature controlled expression of heterologous genesin LAB [158]. Genes for restriction-modification (R/M) systems have also been identified on plasmids pCI65st [159], pSt08, pSt0 [161], and pER35 [164].

Most *S. thermophilus* plasmids replicate via the RCR mechanism. Most RCR plasmids belong to the pC194 family, however pSMQ172 has been assigned to the pE194/pMV158 family [166]. The plasmids pSMQ-316 and pSMQ-312b replicate via the theta-replicating mode [167].

### 2.6. Plasmids of Genus Bifidobacterium

The genus *Bifidobacterium* shares some of the phenotypic traits of genuine LAB, but bifidobacteria are phylogenetically unrelated to other LAB, belonging to the phylum *Actinomyces* branch of bacteria, and have a unique fructose-6-phosphate phosphoketolase pathway of sugar fermentation [1,168,169]. Some *Bifidobacterium* strains naturally colonize the human gastrointestinal tract (GIT) and vagina, are beneficial to health by means of the production of short chain fatty acids and the exclusion of intestinal pathogens, as well as modulation of the immune function [170]. Some *Bifidobacterium* strains are used in the food industry because of their probiotic activities. Therefore researchers show considerable scientific and technological attention to this genus.

Plasmids have been detected in nine of the 31 species of *Bifidobacterium*, including *Bifidobacterium asteroids* [171,172], *Bifidobacterium breve* [173,174], *Bifidobacterium bifidum* [174], *Bifidobacterium catenulatum* [175,176], *Bifidobacterium indicum* [171,172], *Bifidobacterium longum* [177,178,179,180,181,182,183,184,185], *Brevibacterium linens* [186], *Bifidobacterium pseudolongum* subsp. *globosum* [187], and *Bifidobacterium pseudocatenulatum* [188]. Most plasmids are rolling circular plasmids, and their size varies from 1.847–10.22 kb. A few plasmids showed characters of theta replication plasmids [175,176,179,181]. The *ori* region of pSP02 was used to construct a series of first generation cloning vectors able to replicate in many bifidobacterial species [185].

### 2.7. Plasmids of Genus Oenococcus

The genus *Oenococcus* only contained two species *Oenococcus oeni* (formerly called *Leuconostoc oenos*), and *Oenococcus kitaharae*. As its name implies, *O. oeni* plays a pivotal role in the field of oenology. During the fermentation of wine, *O. oeni* is responsible for performing malolactic conversion, an important secondary fermentationin the production of wine [189,190,191].

Several small cryptic plasmids of *O. oeni* have been sequenced and described to date, *i.e.*, pOM1, pLo13, p4028, pOg32, pRS1, pRS2, pRS3, pUBLO1, pUBLO5, and pUBLO6 [192,193,194,195,196,197]. The plasmids pOENI-1 (18.3 kb) and pOENI-1v2 (21.9 kb) are detected in three industrial starters (C9, C10, and C6) and a new isolate S11 [198]. Sequence analyses of plasmids indicate that they carry two genes possibly involved in wine adaptation encoding a predicted sulphite exporter (*tau E*) and a NADH: flavin oxidoreductase [198]. Due to the important role of *O. oeni* in the process of wine-making, various attempts have been made to develop cloning vectors and transformation protocols for *O. oeni* based on these plasmids [192,193,194,195,196,197,198,199,200]. At the beginning, small RCR plasmids pRS1, pRS2 and pRS3 from *O. oeni* were used for development of vectors [196,200]. However, RCR plasmids are less stable than theta-type-replicating plasmids [7]. Therefore, researchers begin to pay attention to the large theta-replicating plasmids from *O. oeni*, for example, pOENI-1 (18.3 kb), pOENI-1v2 (21.9 kb), and pRS7 (~20 kb) [198,201,202]. The plasmid pRS7 from *O. oeni* CT86, is a different plasmid to pOENI-1 and pOENI-1v2 but possesses a *repA* gene homologous to those of the plasmids of the “pOENI-1 family” [202]. Recently, a shuttle vector, pRS7Rep (6.1 kb), was constructed using the replication region of pRS7. The vector pRS7Rep can replicate in *E. coli* and some LAB, including *P. acidilactici*, *L. plantarum*, *L. casei*, *L.citreum*, and *Enterococcus faecalis*. Meanwhile, it contains single restriction sites useful for cloning purposes, and can improve characteristics of some LAB starter strains by means of expression of different exogenous proteins.

*O. kitaharae* was isolated from a composting distilled Shochu residue [203]. Comparing with *O. oeni*, the genome of *O. kitaharae* contains more genes which are involved in cellular defense, such as bacteriocins, antimicrobials, restriction-modification systems and a CRISPR locus [204]. One plasmid was found in *O. kitaharae* DSM 17330 [204].

## 3. Plasmid Replication Mechanisms

In LAB, the most common replication mechanisms are the Sigma and Theta modes of replication. The mode of replication of plasmids has an important impact on some characteristics of plasmid-derived vectors, namely host range, stability, and copy number [7].

### 3.1. Rolling-Circle Replicating Plasmids

The sigma mode of replication, is also named as RCR. RCR plasmids are usually small in size (less than 10 kb), have multiple copies, and are tightly organized. RCR plasmids are ubiquitous in Gram-positive bacteria, although they have been reported in many Gram-negative bacteria and archaea [205].

The common components of RCR plasmids are replication initiator (Rep) protein, the double strand origin (*dso*), and the single strand origin (*sso*). RCR generally involves two main stages, *i.e.*, leading strand replication and lagging strand replication [92,205]. RCR type plasmids replication initiates with specific binding of the plasmid-encoded Rep protein to the cognate *dso*, which contains a specific protein binding sequence, and introduces a cut within the nick site of the *dso*. The leading strand is synthesized at the free 3′OH end at the nick, and produces single-stranded DNA replication intermediates [205,206]. The displaced single strand DNA (ssDNA) is then converted into double strand DNA (dsDNA) through the synthesis of the lagging strand by host proteins initiating at the *sso* [92,205].

Based on the sequence similarities in initiator proteins and *dso*, the RCR plasmids can be classified into several families, e.g., pT181, pE194/pMV158, pC194, and pSN2 family [205,206]. In general, plasmids of the RCR-type from Gram-positive bacteria often have a broad host range.

### 3.2. Theta-Type-Replicating Plasmids

At present six classes of theta replicons have been recognized [90,207,208,209]. Class A includes plasmids which encode a replication protein (*Rep*) and have a characteristic replication origin, designated *oriA*, and are independent of DNA polymerase I (PolI). The *ori* region generally consists of an AT-rich region which usually is followed by three and a half 22 bp direct repeats (called iterons) and two small inverted repeats which overlap the ribosome binding site of the *rep* gene and the -35 site of the *rep* promoter [9,87]. A number of plasmids from Gram-negative hosts are grouped in class A. Most LAB theta-type-replicating plasmids belong to class A, for example, pCI305 [86], pWVO2 [87], pW563 [88], pCD4 [89], and pUCL22 [93] *etc.*

Class B replicons do not encode a Rep protein and lack *oriA*. Their replications are initiated by processing of a transcript synthesized by the host RNA polymerase. Class C replicons, only two of which are known (the closely related plasmids pColE2 and pColE3), encode a replication protein but do not carry an *oriA*-like structure and require PolI for replication. Class D, this replication is dependent on a plasmid-encoded replication protein (Rep) but not on a DNA structure typical for origins of most Rep-dependent plasmids, and is initiated by DNA polymerase I (PolI). pAMβl from *E. faecalis* is recognized as a prototype for class D. Some LAB theta-type-replicating plasmids belong to class D, including the pIP501, pSM19035, and enterococcal plasmid pEF1 [146,210].

The plasmid pLS20 from *Bacillus subtilis* is representative of class E. The plasmid pLS20 shows no similarity with other known plasmid replicons.The structural organization of the pLS20 minimal replicon is entirely different from that of typical rolling circle plasmids from Gram-positive bacteria. The pLS20 minireplicons replicate in *pol*A5 and *rec*A4 *B. subtilis* strains. At same time, pLS20 is different from *Bacillus natto* pLS32 in that the former does not encode a Rep protein [90,209]. Taken together, these results strongly suggest that pLS20 belongs to a new class of theta replicons [209].

Class F, the plasmid pLS32 from *B. natto* was identified as a new family of Gram-positive theta replicons [90]. The family replicons do not possess an AT-rich region, and have a replication initiation protein (RepN), which contains a helix-turn-helix motif. Several iterons are present on RepN proteins, and identified as the origin of replication [73,78,91]. This replication region is structurally dissimilar to those of class A, but has been shown to be DNA polymerase I independent [90]. This family, based on structural organization and homology at the nucleotide and amino acid levels, includes a number of other plasmids of diverse origins, such as *Lactobacillus* plasmids pLJ1 [211], the enterococcal plasmids pAD1 [212,213], pCF10 [214], and pPD1 [215], as well as the lactococcal plasmids pNP40 [73] and pCI2000 [91].

DNA synthesis of theta plasmids can be bidirectional, may initiate from multiple origins; and produces double-stranded DNA replication intermediates, which result in better structural stability [92]. Therefore theta plasmids have a high segregational stability, and can incorporate large DNA fragments as cloning vectors [7].

## 4. Mobility of LAB Plasmids

Plasmids can be transmitted from one bacterium to another (even of another species) via three main mechanisms, *i.e.*, transformation, transduction, and conjugation [216]. Conjugation is a key mechanism for horizontal gene transfer in bacteria [216]. In contrast to conjugation, transformation is a slower process as it requires the existence of free DNA and the achievement of a competent cellular state [217].

The transmissible plasmids can be classified according to their transfer machinery and mobilization ability, *i.e.*, conjugative plasmids and mobilizable plasmids [216,218]. Conjugative (self-transmissible) plasmids contain a self-sufficient conjugative transfer system, which codify all the functions required for their HGT (Horizontal Gene Transfer) [219]. However, mobilizable plasmids carry only a mobilization region (*mob*) encoding specific relaxase and its cognate *oriT*, are incapable of initiating conjugation, and can be transmissible only in the presence of additional conjugative functions provided by either the recipient chromosome or by other auxiliary (alsotermed “helper”) plasmid [220,221]. Therefore conjugative plasmids tend to be large (˃30 kb) with low copy number, while mobilizable plasmids are small (˂15 kb) and have high copy number [222]. The transfer efficiency of mobilizable plasmids is usually lower than that of conjugative plasmids. However, mobilizable plasmids are more frequently found in nature. Therefore mobilizable plasmids have a tremendous impact in horizontal gene transfer in nature [216,221].

Relaxase is an essential ingredient of the MOB machinery, in both conjugative and mobilizable plasmids. It is a key protein that recognizes the origin of transfer (*oriT*), and then initiates and terminates conjugative DNA processing [216]. Small mobilizable plasmids were classified in four main families or super families according to the similarity of their relaxases and the phylogenetic relationships among them [216]. Later the conjugative plasmids were added to this classification system, transmissible plasmids were grouped into six MOB families, *i.e.*, MOB_F_, MOB_H_, MOB_Q_, MOB_C_, MOB_P_, and MOB_V_ [218,222]. The new additional MOB_F_ and the MOB_H_ families are present predominantly in large conjugative plasmids [218]. Most MOB_F_ plasmids are more than 60 kb, and a few of MOB_F_ plasmids are 20–60 kb. All MOB_H_ plasmids are more than 60 kb. The MOB_P_ and MOB_Q_ plasmids are uniformly distributed in plasmids of all size ranges. MOB_C_ is present inmid-sized plasmids (5–60 kb).

Over 50% of MOB_V_ plasmids are less than 5 kb. Most RCR plasmids of Firmicutes belong to the MOB_V_ family. The Mob relaxase from the streptococcal plasmid pMV158 is the representative of the family [220,222]. A lot of relaxases from LAB plasmids are identified in that they belong to the MOB_V_ family, including p141, pAMalpha1, pBM02, pCD034-1, pCD034-2, pF8801, pG6301, pGL2, pK214, pLA106, pLAB1000, pLAC1, pLB4, pLB925A02, pLC88, pM4, pMBLR00, pMRI5.2, pPB1, pPLA4, pS86, pSD11, pSMA23, pTXW, pWCZ, and pYSI8 *etc.* [27,30,31,32,108,220,223,224,225,226]. Most plasmids are small size (˂15 kb), except pPLA4 (8.135 kb), pAMalpha1 (9.759 kb), and pK214 (29.871 kb).

Most plasmids from *B. longum* are classified into the MOB_Q_ family, e.g., pDOJH10L and pDOJH10S from *B. longum* DJO10A [181]; pKJ36 and pKJ50 from *B. longum* KJ [177]; pBLO1 from *B. longum* NCC2705 [178]; pNAC2 from *B. longum* RW041 [179]; p6043A and p6043B from *B. longum* DPC6043; pMG1 and pTB6 [180]. All of plasmids are less than 5 kb and replicate by means of rolling-circle replication.

## 5. Conclusions

In the past 20 years, LAB plasmid biology has become an important area of LAB researches since plasmids associated with many important functions and some industrially relevant characteristics of LAB species; and LAB plasmids may be used to construct new vectors which can change the characters of hosts and accelerate the industrial and biotechnological applications of hosts. At present, a large number of new LAB plasmids have been isolated, characterized, and reconstructed. A series of wide host range vectors have been developed, based on LAB plasmids, and used for transformation of LAB and other hosts. A lot of heterologous proteins have been expressed in LAB by means of LAB plasmid derived vectors. LAB are generally regarded as safe (GRAS) microorganisms, therefore they are used as a delivery vector for therapeutic proteins and antigens, and to develop effective vaccines against potential pathogens and their evolving antibiotic resistance trends. All of these advancements will accelerate the developments of LAB plasmids and LAB.

## Supplementary Materials

Supplementary materials can be found at http://www.mdpi.com/1422-0067/16/06/13172/s1.
