# Supplementary material for: Plasmids from Food Lactic Acid Bacteria: Diversity, Similarity, and New Developments
Source: Int J Mol Sci. 2015 Jun 10;16(6):13172–202. doi: 10.3390/ijms160613172 (PMC4490491; doi:10.3390/ijms160613172)
Supplement: Supplementary file 1 [file ijms-16-13172-s001.pdf]

Supplmentary Information

Table S1. The plasmids of *Lactobacillus plantarum*.

Number	Plasmid Name	Strain	Replication	Size (Kb)	(G + C)%	Protein	Gene	Pseudogene	Rep Protein	Mob Protein	Function	Accession Number	Reference	Ssource
1	p256	*L. plantarum* NC7	theta	7.22	36.7	10	10	–			A toxin–antitoxin-likeplasmid maintenance system	NC_006278.1	[40]	
2	pA1	*L. plantarum* A112	RCR	2.82	35	2	2	–	RepA 47 aa<br>RepB 196 aa			NC_010098.1	[227]	
3	pC30il	*L. plantarum*	RCR	2.14	37	1	1	–	Rep 317 aa			NC_001370.1	[228]	
4	pCMPG5300.01	*L. plantarum* CMPG5300		2.13	38	2	2	–	Rep 318 aa			CM002919.1	[229]	Vagina of healthy woman
5	pCMPG5300.02			10.94	38.6	8	9	1	Rep 309 aa		Type IIrestriction/modification system; DNA helicase, YeeB; Toxin-antitoxin system, PemK/MazF family	CM002920.1		
6	pCMPG5300.03			27.84	40.5	32	34	2	RepA 373 aa	Mob 687 aa	Antitoxin, RelB/DinJ family; Site-specific recombinase; Pyridine nucleotide-disulfide oxidoreductase; Conjugation protein; Thioredoxin family protein	CM002921.1		
7	pG6301	*L. plantarum* G63	RCR	3.52	37.3	2	2	–	Rep 318 aa	Mob 361 aa		NC_019372.1	[32]	Chinese pickle
8	pG6302		theta	9.11	36.4	14	14	–	RepB 200 aa			NC_019379.1		
9	pG6303			10.05	36.8	10	10	–			Cytochrome b; Stress induced DNA binding protein	NC_019371.1		
10	pLBPp1	*L. plantarum* subsp. *plantarum* P-8		51.18	39.4	57	58	1	RepA 300 aa		Nickase; DNAtopoisomerase; Cold shock protein; Prophage protein	NC_021233.1	[230]	
11	pLBPp2			46.58	42.2	42	44	2			Collagen adhesionrotein; 6-phospho-β-glucosidase; Galactosidase; Nisin resistance protein; Potassium uptake protein	NC_021225.1		
12	pLBPp3			39.47	42.3	44	44	–	RepB 303 aa		Succinatedehydrogenase/fumaratereductase; Peptidoglycan-binding protein; Aspartate aminotransferase	NC_021226.1		

Table S1. *Cont.*

Number	Plasmid Name	Strain	Replication	Size (Kb)	(G + C)%	Protein	Gene	Pseudogene	Rep Protein	Mob Protein	Function	Accession Number	Reference	Ssource
13	pLBPp4	*L. plantarum* subsp. *plantarum* P-8		30.69	39.8	27	27	–			Multidrug efflux pump protein; Transposase; Potassium uptake protein; Cold shock protein;nickase	NC_021234.1	[230]	
14	pLBPp5			16.11	42.1	18	18	–	RepB 62 aa	MobB 201 aa MobC 197 aa	Cation-transporting ATPase	NC_021227.1		
15	pLBPp6			8.69	36	10	10	–	RepA 311 aa RepB 182 aa		Stress induced DNA binding protein; Insertion element	NC_021228.1		
16	pLD1	*L. plantarum* KLDS 1.0801	RCR	2.11	37.8	1	1	–	Rep318 aa			NC_012220.1		
17	pLFE1	*L. plantarum* M345	RCR	4.03	34.4	5	5	–	RepA 59 aa RepB 211 aa	Mob 83 aa	Erythromycin	NC_012628.1	[24]	
18	pLJ42	*L. plantarum*	theta	5.53	42.4	4	4	–	RepA 308 aa	MobC 112 aa MobA 445 aa MobB 229 aa		NC_019219.1		
19	pLp16A	*L. plantarum* 16		7.24	33.4	2	2	–	Rep 311 aa			NC_021515.1	[20]	Malt production steep water
20	pLp16B			8.64	35.9	2	2	–	RepA 311 aa		Peptidoglycan binding domain-containing protein	NC_021525.1		
21	pLp16C			27.28	39.9	32	32	–	RepA 366 aa	MobA 376 aa	Conjugation protein TrsK	NC_021516.1		
22	pLp16D			37.1	40.8	38	38	–	Rep 288 aa		Stress-responsive transcriptional regulator; TraA-like nicking enzyme; Cell-wall-anchored protein	NC_021526.1		
23	pLp16E			40.15	41.4	41	41	–	Rep 367 aa			NC_021517.1		
24	pLp16F			50.19	39.8	57	57	–	RepB 286 aa		Chloride channel protein; Na^+/H^+ antiporter NapA; Transposase; Potassium uptake protein; Glycine/betaine/carnitine ABC transporter	NC_021518.1		
25	pLp16G			51.86	42.6	52	52	–	RepE 512 aa Rep 283 aa		Conjugation-related ATPase; DNA topoisomeras malt production steep watere; IS3 family transposase	NC_021527.1		

Table S1. *Cont.*

Number	Plasmid Name	Strain	Replication	Size (Kb)	(G + C)%	Protein	Gene	Pseudogene	Rep Protein	Mob Protein	Function	Accession Number	Reference	Ssource
26	pLp16H			74.08	41.5	74	74	–	RepB 257 aa<br>Rep 510 aa		Glucose-1-phosphate thymidylyltransferase; Exopolysaccharide biosynthesis protein; Polysaccharide biosynthesis protein	NC_021519.1		
27	pLp16I			13.34	37.9	8	8	–	Rep 307 aa	MobA 269 aa<br>Mob 230 aa		NC_021528.1		
28	pLp16L			6.46	34.7	4	4	–			Cell surface protein	NC_021520.1		
29	pLP18	*L. plantarum* PC518	RCR	1.81	37.5	2	2	–	RepA 237 aa			NC_014627.1	[15]	Chinese pickle
30	pLP2000	*L. plantarum* AS1.2986	RCR	2.06	38.2	1	1	–	Rep 317 aa			NC_003893.1	[231]	
31	pLP2111	*L. plantarum* CICC 6002		2.11	38.3	1	1	–	Rep 317 aa			NC_011497.1		
32	pLP2140	*L. plantarum* S1		2.14	38.3	1	1	–	Rep 393 aa			NC_013541.1		
33	pLP9000	*L. plantarum* AS1.2986	RCR	9.25	37.4	9	9	–				NC_003894.1	[231]	
34	pLP-ZJ101	*L. plantarum* ZJ316		15.17	40.2	17	17	–	RepA 311 aa<br>RepA 98 aa	Mob 389 aa<br>MobC 117 aa<br>MobC 113 aa	Calcium-transporting ATPase	NC_021903.1	[232]	Healthy infant fecal samples
35	pLP-ZJ102			39.12	38.7	53	53	–	RepA 97 aa		UDP-glucose 4-epimerase; Galactosyltransferase; Glycosyltransferase; Glycerol-3-phosphate cytidylyltransferase	NC_021904.1		
36	pLP-ZJ103			41.51	39.5	47	47	–			Ribonucleoside-diphosphatereductase	NC_021912.1		
37	pLR1	*L. plantarum* LR1	RCR	2.07	37.8	2	2	–	RepA 318 aa			NC_011136.1	[233]	Sichuan pickle
38	pLTK13	*L. plantarum* L137		34.52	39	25	25	–	RepA102 aa<br>RepB 246 aa		Amylopullulanase; Nickase; Permease; Anaerobicribonucleoside triphosphate reductase	NC_011101.1	[234]	Plant traditional fermented food
39	pLTK2	*L. plantarum* L137		2.3	38.6	1	1	–	RepA 317 aa			NC_002123.1	[235]	
40	pM4	*L. plantarum* M4	RCR	3.32	38.7	3	3	–	Rep 318 aa	Mob 362 aa		NC_009666.2	[31]	Fresh milk

Table S1. *Cont.*

Number	Plasmid Name	Strain	Replication	Size (Kb)	(G + C)%	Protein	Gene	Pseudogene	Rep Protein	Mob Protein	Function	Accession Number	Reference	Ssource
41	pMD5057	*L. plantarum* 5057	theta	10.88	36.2	9	10	1	RepB 172 aa<br>RepA 311 aa		Tetracycline resistance	NC_004944.1	[22]	Corn silage
42	pMRI 5.2	*L. plantarum* BFE 5092	RCR	5.21	35.8	7	7	–	Rep 314 aa<br>RepA 52 aa<br>RepB 215 aa<br>RepC 48 aa	Mob 408 aa		NC_019900.1	[30]	Fermented milk
43	pPB1	*L. plantarum* BIFI-38	RCR	2.9	37.7	4	5	–	RepB 208 aa	Mob 355 aa		NC_006399.1	[224]	Wine
44	pPLA4	*L. plantarum* 423		8.35					RepB 270 aa	Mob 361 aa	Bacteriocin 423	NG_036264.1	[236]	
45	pR18	*L. plantarum* PA18	RCR	3.21	35.8	2	2	–	RepA 317 aa		Lincosamide nucleotidyltransferase	NC_019321.1	[33]	Plant
46	pST-III	*L. plantarum* subsp. *plantarum* ST-III	theta	53.56	38.7	42	45	3	RepB 286 aa		A high-affinity K^+-transport system	NC_014558.2	[25]	Chinese pickle
47	pLP1	*L. plantarum* CCM1904							Rep 317 aa				[237]	
48	pLB4	*L. plantarum* NCDO1088							Rep 52 aa	Mob 361 aa			[238]	
49	p8014-2	*L. plantarum* ATCC 8014	RCR						Rep 312 aa				[239]	
50	pWCFS101	*L. plantarum* WCFS1	RCR	1.92	39.5	3	3	–	RepA 319aa			NC_006375.1	[28]	Human saliva
51	pWCFS102		RCR	2.36	34.3	4	4	–	RepA 52 aa<br>RepB 219 aa			NC_006376.1		
52	pWCFS103		theta	36.07	40.8	43	43	–	Rep A 102 aa		Repressor of arsenical resistance;<br>Arsenical pump-driving ATPase	NC_006377.1		
53	pXY3	*L. plantarum* XY3	RCR	2.97	38.2	7	7	–	Rep 219 aa		Recombinase	NC_013789.1	[29]	Koumiss
54	pZL2	*L. plantarum* subsp. *Plantarum* Zhang-11		3.26	38.2	2	2	0	Rep 318 aa	Mob 361 aa		NC_024985.1		Fermented rice
55	pZL3			11.9	37.7	15	15	0	RepB 311 aa<br>RepA 304 aa	MobA 23 aa	Class II bacteriocin	NC_024984.1		
56	pZL4			12.52	39.4	11	11	0	RepA 311 aa<br>RepB 172 aa		Type I restriction-Modification protein subunit M; Toxin-antitoxin system;<br>Tyrosine recombinase Int;<br>Glycerol-3-phosphate transporter	NC_024988.1		

Table S2. The plasmids of *Lactococcus latctis*.

Number	Plasmid Name	Stain	Replication	Size (Kb)	(G + C)%	Protein	Gene	Pseudogene	Rep Protein	Mob Protein	Function	Accession Number	Reference	Niche
1	pAF04	*L. lactis* DPC3758	Theta	3.8	32	4	4	–	RepB 402 aa	MobC 122 aa		NC_019347.1	[61]	Raw milk cheeses
2	pAF07		Theta	7.44	36.4	6	6	–	RepA 296 aa	MobC 123 aa<br>MobA 320 aa		NC_019348.1		
3	pAF12		Theta	12.07	*33.3*	11	12	–	RepB 441 aa	MobC 122 aa<br>MobD1 135 aa<br>MobD2 376 aa	Type I restriction-modification HsdS subunit; MaturaseMatR	NC_019349.1		
4	pAF14		Theta	14.42	34.1	11	13	–	RepB 440 aa	MobC1 164 aa<br>MobA 410 aa<br>MobB 207 aa<br>MobC2 200 aa	Cation transport ATPase; Cadmium efflux ATPase CadA; Cadmium resistance regulator CadC	NC_019350.1		
5	pAF22		Theta	22.39	34.9	23	23	–	RepB 388 aa		Serine Recombinase family; Conjugation protein	NC_019351.1		
6	pAG6	*L. lactis* 712	Theta	8.66	33.7	8	8	–	RepB 385 aa		Cadmium resistance protein	NC_007191.1		
7	pAH33	*L. lactis* DPC220	Theta	6.16	35.9	7	7	–	RepB 386 aa		Type I R/M system, R/M82; Magnesium transporter CorA; Cytochrome B; Type I specificity subunit; HsdS	NC_002150.1	[72]	
8	pAH82		Theta	20.33	34.4	17	17	–	RepB 386 aa		Phage resistance; Cadmium resistance regulator CadC; Serine/threonine protein phosphatase; Type I R/M system, R/M 82	NC_004966.1		
9	pAH90	*L. lactis*	Theta	26.49	34.8						Phage resistance; Type I specificity subunits, hsdSI, hsdSII; Cadmium resistance; Magnesium/cobalt transporter, corA		[240]	
10	pAR141	*L. lactis* M14	RCR	1.59	36.1	2	2	–	RepB 192 aa			NC_013783.1	[103]	
11	pAW153	*L. lactis* W15	Theta	7.12	31.4	8	8	–	RepB 391 aa<br>RepX 154 aa		restriction-modification (R/M) system LlaCI	NC_017494.1	[71]	
12	pBL1	*L. lactis* IPLA 972	Theta	10.9	32.6	8	8	–	RepB 385 aa		Lactococcin 972 production, lclA Bacteriocin immunity, lclB	NC_004955.1	[57]	
13	pBM02	*L. lactis* P8-2-47	RCR	3.85	35.7	6	6	–	RepA 49 aa<br>RepB 213 aa	Mob-like 304 aa		NC_004930.1	[102]	

Table S2. *Cont.*

Number	Plasmid Name	Stain	Replication	Size (Kb)	(G + C)%	Protein	Gene	Pseudogene	Rep Protein	Mob Protein	Function	Accession Number	Reference	Niche
14	pCD4	*L. lactis* MJC15	Theta	6.09	33.4	5	6	–	RepB 386 aa		Type I specificity subunit; HsdS tyrosine family DNA recombinase OrfA	NC_002748.1	[89]	
15	pCI2000	*L. lactis* subsp. *lactis* 275	Theta						RepA 391 aa			NG_035219.1	[91]	
16	pCI305	*L. lactis* UC317	Theta	8.69	32.4	8	8	–	RepB 386 aa		Type I specificity subunit, hsdS	NC_002502.1	[86]	
17	pCI528	*L. lactis* subsp. *Cremoris* UC503	Theta						Rep 383 aa		Type I restriction enzyme specificity protein	AF013595.1	[241]	
18	pCIS1	*L. lactis* subsp. *cremoris* UC509.9	Theta	4.26	32	2	2	–	RepB 383 aa		Type I restriction enzyme specificity protein	NC_019438.1	[55]	Irish dairy starter.
19	pCIS2		Theta	5.46	30.1	5	6	–	RepB 400 aa	MobC 122 aa	Putative cytochrome B	NC_019434.1		
20	pCIS3		Theta	6.16	35.9	4	5	–	RepB 386 aa		Defense mechanisms; Methylase_S; Mg^{2+} and Co^{2+} transporter; Inorganic ion transport and metabolism; Family: CorA	NC_019433.1		
21	pCIS4		Theta	7.05	38.4	8	9	–	RepA 309 aa<br>RepB 163 aa	MobC 122 aa	Signal transduction mechanisms (protein-tyrosine-phosphatase); ABC transporter	NC_019437.1		
22	pCIS5		Theta	11.68	34.1	11	12	–	RepB 309 aa<br>OrfX 269 aa		IS6 family transposase; Putative regulatory protein; Coenzyme transport and metabolism; IS982 family transposase	NC_019432.1		
23	pCIS6		Theta	38.67	37.1	27	30	–	RepB 381 aa<br>OrfX 189 aa<br>RepC 76 aa	MobD 505 aa<br>MobC 161 aa	Glycopeptide antibiotics resistance protein; IS6 family transposase; Transposase; amidase/aminoacylase; Peptidase E; Pyrrolidone-carboxylate peptidase	NC_019436.1		
24	pCIS7		Theta	53.05	32.4	41	50	–	RepB 450 aa<br>OrfX 196 aa	MobC 122 aa	IS982 family transposase; Cell cycle control; IS6 family transposase; Amino acid transport and metabolism (para-aminobenzoate synthase component II); Carbohydrate transport and metabolism (permease, major facilitator superfamily)	NC_019431.1		

Table S2. *Cont.*

Number	Plasmid Name	Stain	Replication	Size (Kb)	(G + C)%	Protein	Gene	Pseudogene	Rep Protein	Mob Protein	Function	Accession Number	Reference	Niche
25	pCIS8		Theta	80.59	34	67	79	–	RepB 385 aa<br>RepB385 aa<br>RepB 383 aa	MobA 673 aa	IS6 family transposase; Mn^{2+}/Fe^{2+} transporter; Universal stress protein	NC_019430.1		
26	pCL2.1	*L. lactis* ML8	RCR	2.05	34	2	3	–	RepB 203 aa			NC_004981.2	[100]	
27	pCRL1127	*L. lactis* CRL1127	Theta	8.28	34.8	7	7	–	RepB 385 aa		Citrate permease plasmid; Putative translational regulators, citQ, citR	NC_003101.1		
28	pCRL291.1	*L. lactis*	Theta	4.64	33.51	3	3	–				NC_002799.1		
29	pCV56A	*L. lactis* subsp. *lactis* CV56	Theta	44.1	32.1	39	41	2	RepB 386 aa	MobC 122 aa<br>MobD 503 aa	Type II restriction-modification system; Acetyltransferase; DNA RepAir ATPase; Recombinase A	NC_017483.1	[62]	Vaginas of Healthy Women
30	pCV56B		Theta	35.93	34.5	28	33	5	RepB 383 aa<br>RepX 196 aa	MobC1 121 aa<br>MobA 410 aa<br>MobC2 200 aa	type I restriction-modification system; Mg^{2+} and Co^{2+} transporter; DNA-invertase /resolvase; Amidase; Oxalate/FormateAntiporter	NC_017487.1		
31	pCV56C		Theta	31.44	32.5	29	31	2	RepB 1392 aa<br>RepX 253 aa	MobD 505 aa<br>MobC 122 aa	Conjugation protein; Resolvase; Copper-potassium Transporting ATPase B; Cytochrome B	NC_017484.1		
32	pCV56D		Theta	5.54	32.2	8	8	–	RepB 386 aa<br>RepX 118 aa		Cytochrome B	NC_017485.1		
33	pCV56E		RCR	2.26	33.8	3	3	–	RepA 232 aa<br>RepC 49 aa			NC_017488.1		
34	pDBORO	*L. lactis* DB0410	Theta	16.4	35.2	15	15	–	RepB 383 aa		The toxic pyrimidine analogue 5-fluoroorotate	NC_009137.1	[67]	
35	pDI25	*L. lactis* subsp. *lactis* 5136	RCR	5.5kb					Rep 233 aa				[98]	
36	pDR1-1	*L. lactis*	Theta	7.41	33.7	6	6	–	RepB 423 aa		Type I R/M system specificity subunit	NC_004164.2	[242]	
37	pDR1-1B	*L. lactis* DRC1	Theta	7.34	33.74	6	6	–	RepB 386 aa		Type I specificity subunit, hsdS	NC_004163.1	[242]	

Table S2. *Cont.*

Number	Plasmid Name	Stain	Replication	Size (Kb)	(G + C)%	Protein	Gene	Pseudogene	Rep Protein	Mob Protein	Function	Accession Number	Reference	Niche
38	pFV1201	*L. lactis* subsp. *cremoris* KH (MG1614)	Theta						RepB 385 aa			NG_034745.1	[243]	
39	pGdh442	*L. lactis* NCDO1867	Theta	68.32	35.1	63	67	1	RepB 450 aa	MobC 122 aa	GlutamateDehydrogenase; Orotate transporter	NC_009435.1	[66]	
40	pHP003	*L. lactis* HP	Theta	13.43	40.1	6	6	–	RepB 385 aa RepC 76 aa		Lactocepin genes, prtP, prtM	NC_004847.1	[75]	
41	pIL1	*L. lactis* IL594	Theta	6.38	32.3	7	7	–	RepB 385 aa		Type I S-subunit protein; IS-like element; Citrate permease/transporter	NC_015860.1	[63]	
42	pIL2		Theta	8.28	34.8	10	10	–	RepB 402 aa		Resolvase; Major facilitator superfamily permease; Multicopperoxidase; Nicotinamide mononucleotide transporter	NC_017489.1		
43	pIL3		Theta	19.24	35.1	20	20	–	RepB 394 aa	MobC 122 aa	Transposase; Na^{+}/H^{+} antiporter; K^{+} transporter; K^{+} transport system protein	NC_015861.1		
44	pIL4		Theta	48.98	35.1	47	50	–	RepB1 384 aa RepB 388 aa	MobC 123 aa MobD 505 aa	Peptidase E; Cadmium resistance regulator CadC; Cadmium efflux ATPase CadA; Glycopeptide antibiotics resistance protein	NC_015862.1		
45	pIL5		Theta	23.4	34.5	22	22	–	RepB 383 aa RepX 206 aa	MobA 410 aa MobB 207 aa MobC 200 aa	Type I R/M system restriction subunit; Type I R/M system methylation subunit; Cold shock proteins C and D	NC_015863.1		
46	pIL6		Theta	28.43	33.6	25	27	–	RepB 445 aa	MobC 122 aa MobD 503 aa	Type I S-subunit protein; IS-like element; Citrate permease/transporter	NC_019308.1		
47	pIL7		Theta	28.55	34.1	26	28	–	RepB 383 aa RepC 76 aa RepA 450 aa	MobC 122 aa MobD 505 aa	HsdSsubunit;dynamin; IS-LL6 transposase/hypothetical protein; 2-dehydropantoate 2-reductase; Type I restriction enzyme R protein	NC_015864.1		
48	pIL103	*L. lactis* IL964	Theta						RepB 383 aa		Type Ic restriction-modification system	NG_034966.1	[244]	
49	pIL105	*L. lactis* IL964	Theta	8.506	29.8	7	7	–	RepB 402 aa		Phage abortive infection gene, abiD1	NC_000906.2	[245]	
50	pIL2614	*L. lactis* IL1403	Theta	10.066					RepB 386 aa		Type IC restriction subunit	NG_034933.1	[246]	

Table S2. *Cont.*

Number	Plasmid Name	Stain	Replication	Size (Kb)	(G + C)%	Protein	Gene	Pseudogene	Rep Protein	Mob Protein	Function	Accession Number	Reference	Niche
51	pJW563	*L. lactis* subsp. *Cremoris* W56 (MG1614)	Theta	11.5					RepB 385 aa			NG_034688.1	[88]	
52	pJW566		Theta						RepB 389 aa			NG_034747.1		
53	pK214	*L. lactis* K214	Theta	29.87	32.4	29	33	2	RepB 384 aa	Mob 403 aa	Multidrug transport protein, mdtA Streptomycin resistance, str; Chloramphenicol resistance, cat; Tetracycline resistance, tetS	NC_009751.1	[247]	
54	pKF147A	*L. lactis* subsp. *lactis* KF147	Theta	37.51	32.4	29	38	9	RepA 450 aa		Metal-dependent membrane protease; XRE family transcriptional regulator; IS6/IS1216E family transposase; Potassium transport system protein	NC_013657.1	[68]	Mung bean sprouts
55	pKL001	*L. lactis* KLDS4.0319-3	Theta	6.07	32.9	4	4	–	RepB 355 aa		Thymidylate kinase; Orotidine-5-phosphate decarboxylase	NC_011610.1		
56	pKP1	*L. lactis* BGKP1	Theta	16.18	35.9	7	7	–	RepB 386 aa RepX 195 aa		Pyrrolidone-carboxylate peptidase; Mucin-binding domain-containing protein; Bacterial cell aggregation	NC_016042.1	[248]	Artisanal semi-hard homemade cheese
57	pL2	*L. lactis*	Theta	5.3	32.5	5	5	–	RepB 386 aa			NC_008594.1	[249]	
58	pLP712	*L. lactis* NCDO712	Theta	55.4	37.4	44	61	17	RepC 76 aa RepA 450 aa		Encode genes for lactose catabolism and a serine proteinase involved in casein degradation	NC_019377.1	[64]	
59	pMN5	*L. lactis* BGMN1-5	RCR	5.67	30.3	4	4	–	RepA 316 aa		GroupIIcbacteriocin genes, lsbA, lsbBMultidrug transporter, lmrB;	NC_004922.1	[58]	
60	pMRC01	*L. lactis* DPC3147	Theta	60.23	30.11	63	64	–	RepB 384 aa		Phage resistance, Lacticin 3147	NC_001949.1	[56]	
61	pNCDO2118	*L. lactis* subsp. *lactis* NCDO 2118	Theta	37.571	32.3	32			RepA 450 aa			NZ_CP009055.1	[96]	Frozen peas
62	pND302	*L. lactis* M71	Theta						RepB 385 aa			NG_034801.1	[60]	
63	pND324	*L. lactis* M127	Theta	3.6	33.4	3	3	–	RepB 383 aa RepC 76 aa			NC_008436.1	[77]	

Table S2. *Cont.*

Number	Plasmid Name	Stain	Replication	Size (Kb)	(G + C)%	Protein	Gene	Pseudogene	Rep Protein	Mob Protein	Function	Accession Number	Reference	Niche
64	pND608	*L. lactis* FG2	Theta						Rep 386 aa			U26550.1	[250]	
65	pNP40	*L. lactis* DRC3	Theta	64.98	32.3	62	62	–	RepA 398 aa<br>RepB 282 aa		Bacteriophage resistance systems	NC_010901.1	[73]	
66	pNZ4000	*L. lactis*<br>NIZO B40	Theta	42.81	33.3	45	45	–	RepB1 399 aa<br>RepB2 401 aa<br>RepB3 383 aa<br>RepB4 388 aa		Exopolysaccharide biosynthesis, Eps gene cluste<br>Magnesium/cobalt transporter,<br>corA conjugative transfer region	NC_002137.1	[70]	
67	pQA504	*L. lactis* subsp. *cremoris* A76	Theta	3.98	37.8	3	3	–	Rep 508 aa	Mob 449 aa		NC_017497.1	[95]	Cheese production
68	pQA518		Theta	17.66	37.4	12	12	–	Rep 388 aa		DNA restrictase	NC_017495.1		
69	pQA549		Theta	49.22	35.1	42	42	–	Rep 384 aa<br>Rep 391 aa		Serine protease	NC_017493.1		
70	pQA554		Theta	53.63	34.9	69	69	–	Rep 450 aa			NC_017496.1		
71	pS7a	*L. lactis* S50	Theta	7.301	33.4	5	5	–	RepB 383 aa		Type I specificity subunit, hsdS	NC_004652.1	[76]	
72	pS7b		Theta	7.263	33.65	5	5	–	RepB 421 aa		Type I specificity subunit, hsdS	NC_004653.1		
73	pSH71	*L. lactis* subsp. *cremoris*	RCR									A09338.1	[97]	
74	pSK11A	*L. lactis* SK11	Theta	10.37	30.9	13	14	–	RepB 385 aa<br>RepX 214 aa	MobC 122 aa	Serine/threonine phosphatase family protein	NC_017498.1	[65]	Cheese making
75	pSK11B		Theta	13.33	34.3	14	15	–	RepB1 388 aa<br>RepX1 200 aa<br>RepX1 178 aa<br>RepB2 386 aa		Putative DNA segregation ATPase;<br>α-acetolactate decarboxylase	NC_013551.1		
76	pSK11L		Theta	47.17	34.8	40	45	1	RepB1 384 aa;<br>RepB2 391 aa	MobA 673 aa	Serine protease NisP	NC_017478.1		
77	pSK11P		Theta	75.81	35.4	61	83	2	RepB 388 aa<br>RepX 192 aa<br>RepC 76 aa		EPS biosynthesis protein; Pyrrolidone-carboxylate peptidase; Multicopper oxidase family protein	NC_017500.1		

Table S2. *Cont.*

Number	Plasmid Name	Stain	Replication	Size (Kb)	(G + C)%	Protein	Gene	Pseudogene	Rep Protein	Mob Protein	Function	Accession Number	Reference	Niche
78	pSL2	*L. lactis* ssp. *Lactis* biovar. Diacety *lactis* Bu2	Theta						RepB 385 aa			NG_034642.1	[251]	
79	pSRQ700	*L. lactis* DCH-4	Theta	7.78	34.2	9	9	–	RepB 438 aa		Type II R/M system, LlaDCHI; Type I specificity subunit, hsdS	NC_002798.1	[252]	
80	pSRQ800		Theta	7.86	31.3	7	7	–	RepB 401 aa		Abortive infection gene, abiK; Type I specificity subunit, hsdS	NC_004960.1		
81	pSRQ900		Theta	10.84	31.1	11	11	–	RepB 386 aa		Abortive infection gene, abiQ; Type I specificity subunit, hsdS	NC_004959.1		
82	pUCL22	*L. lactis* Z270	Theta						RepA 388 aa RepB 119 aa			X60454.1	[94]	
83	pVF18	*L. lactis* subsp. *Lactis* bv. *diacetylactis str.* DPC3901	Theta	18.98	33.9	21	25	4	RepB 385 aa		IS1216 transposase; Type I restriction-modification system	NC_015900.1	[69]	Raw milk cheese
84	pVF21		Theta	21.73	33.6	14	16	2	RepB 383 aa Rep 209 aa		Type I restriction-modification system; Glutamate dehydrogenase; Cadmium efflux ATPase CadA	NC_015912.1		
85	pVF22		Theta	22.17	35.1	19	21	2	RepB 441 aa Rep 345 aa	MobC1 164 aa MobA 410 aa MobB 207 aa MobC2 200 aa	Mg^{2+} and Co^{2+} transport protein corA1; Tetracycline resistance protein; Cytochrome B	NC_015901.1		
86	pVF50		Theta	53876	34.5%	52	52		RepA 450 aa	MobC 148 aa MobD 170 aa	IS116/IS110/IS902 family transposase; Cation transport ATPase; Copper chaperone; FNR family transcriptional regulator; Oligopeptide ABC transporter; Sodium/hydrogen exchanger family	NC_015902.1		
87	pVS40	*L. lactis*	Theta						Rep 399 aa			L02920.1	[253]	
88	pWC1	*L. lactis* 2204.	RCR	2.85	29.5	1	1	–	Rep 316 aa			NC_004980.1	[101]	
89	pWVO1	*L. lactis*	RCR	2.18	33.4	4	4	–	Rep 232 aa			NC_002192.1	[99]	
90	pWVO2	*L. lactis* Wg2	Theta	3.83	31.3	1	1	–	RepB 383 aa		Cryptic	NC_002193.1	[87]	

Table S2. *Cont.*

Number	Plasmid Name	Stain	Replication	Size (Kb)	(G + C)%	Protein	Gene	Pseudogene	Rep Protein	Mob Protein	Function	Accession Number	Reference	Niche
91	pWVO4	*L. lactis* Wg2	Theta						RepB 401aa			CAA80965.1	[107]	
92	pWVO5		Theta						RepB 381 aa			CAA80966.1		
93	1	*L. lactis* subsp. *cremoris* SK11	Theta	14.04	34.4	10	11	1	Rep 386 aa<br>Rep 296 aa	Mob 322 aa	Lactococcin A; Bacteriocin-processing peptidase; Nucleoid DNA-binding protein	NC_008503.1	[165]	
94	2		Theta	9.55	30.4	6	8	2	Rep 385 aa		Diadenosinetetraphosphatase-like protein; Transposase; Response regulator	NC_008504.1		
95	3		Theta	74.75	35.4	61	66	3	Rep 388 aa<br>RepB 174 aa	Mob 234 aa<br>MobC 122 aa	Multicopper oxidase; Lactocepin I aminodeoxychorismate synthase; MaturaseMatR; Exopolysaccharide biosynthesis protein; Mg^{2+} and Co^{2+} transporter	NC_008505.1		
96	4		Theta	47.21	34.8	35	39	4	Rep 384 aa		Cellobiose-specific PTS system; Tagatose-6-phosphate kinase; Lactose transport regulator; Oligopeptidase F; Carbon starvation protein	NC_008506.1		
97	5		Theta	14.21	33.5	8	8	–	Rep 443 aa		Superfamily II DNA/RNA helicase	NC_008507.1		
98	1	*L. lactis* subsp. *lactis* KLDS 4.0325	Theta	4.09	30	6	6	–				NC_022587.1	[254]	Home-made koumiss
99	2		Theta	0.87	32.6	2	2	–				-		
100	3		Theta	1.28	32.6	3	3	–	RepB 232 aa			-		
101	pCIS3	*L. lactis* UC509.9	Theta	6.16	35.9	3	4	–	RepB 386 aa		Magnesium/cobalt transporter, corAType I specificity subunit, hsdS	NC_002138.1	[255]	
